# AI-based nanotoxicity data extraction and prediction of nanotoxicity

**DOI:** 10.1016/j.csbj.2025.03.052

**Published:** 2025-04-03

**Authors:** Eunyong Ha, Seung Min Ha, Zayakhuu Gerelkhuu, Hyun-Yi Kim, Tae Hyun Yoon

**Affiliations:** a Department of Chemistry, Hanyang University, Seoul 04763, Republic of Korea; b Research Institute for Convergence of Basic Science, Hanyang University, Seoul 04763, Republic of Korea; c Institute of Next Generation Material Design, Hanyang University, Seoul 04763, Republic of Korea; d NGeneS Inc., Ansan-si 15495, Republic of Korea; e Yoon Idea Lab. Co. Ltd., Seoul 04763, Republic of Korea

**Keywords:** Nanotoxicity, Large Language Models, Data extraction, Prompt engineering, LangChain, Automated machine learning

## Abstract

With the growing use of nanomaterials (NMs), assessing their toxicity has become increasingly important. Among toxicity assessment methods, computational models for predicting nanotoxicity are emerging as alternatives to traditional in vitro and in vivo assays, which involve high costs and ethical concerns. As a result, the qualitative and quantitative importance of data is now widely recognized. However, collecting large, high-quality data is both time-consuming and labor-intensive. Artificial intelligence (AI)-based data extraction techniques hold significant potential for extracting and organizing information from unstructured text. However, the use of large language models (LLMs) and prompt engineering for nanotoxicity data extraction has not been widely studied. In this study, we developed an AI-based automated data extraction pipeline to facilitate efficient data collection. The automation process was implemented using Python-based LangChain. We used 216 nanotoxicity research articles as training data to refine prompts and evaluate LLM performance. Subsequently, the most suitable LLM with refined prompts was used to extract test data, from 605 research articles. As a result, data extraction performance on training data achieved F1_D.E._ (F1 score for Data Extraction) ranging from 84.6 % to 87.6 % across different LLMs. Furthermore, using the extracted dataset from test set, we constructed automated machine learning (AutoML) models that achieved F1_N.P._ (F1 score for Nanotoxicity Prediction) exceeding 86.1 % in predicting nanotoxicity. Additionally, we assessed the reliability and applicability of models by comparing them in terms of ground truth, size, and balance. This study highlights the potential of AI-based data extraction, representing a significant contribution to nanotoxicity research.

## Graphical Abstract

**Figure:**
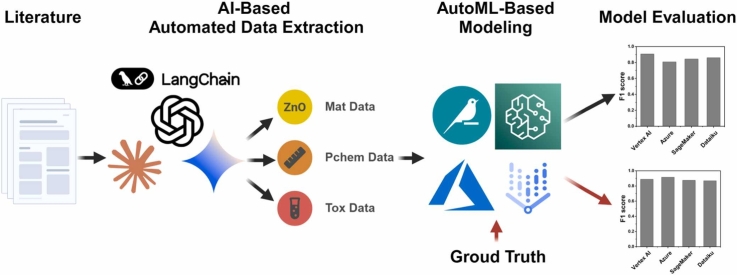

## 1 Introduction

The rise of artificial intelligence (AI) has opened a new chapter in scientific research, with AI becoming a pivotal tool across various disciplines. In nanotoxicity research, the synergy between AI and the evaluation of nanomaterials (NMs) presents significant potential for advancements [1]. AI-powered tools can help researchers manage labor-intensive tasks such as literature reviews, data analysis, and the screening of NMs. The integration of AI into nanotoxicity research offers great potential to deepen our understanding of the toxicity risks associated with NMs and to develop strategies for designing safer nanomaterials and products.

As the use of NMs continues to increase, understanding the relationship between the properties of NMs and their toxicity has become crucial [2]. In recent years, in silico methods have gained significant attention in nanotoxicity research, particularly for developing nanoscale structure-activity relationships (nano-SARs) which identify correlations between the properties of NMs and their toxicity endpoints [3], [4], [5], [6], [7], [8], [9]. This data-driven approach holds the potential to replace traditional in vivo and in vitro assays [10], [11], [12], which are time-consuming, costly, and often raise ethical concerns. In response, the demand for high-throughput, cost-effective screening has driven the advancement of computational modeling in nanotoxicity assessment, particularly through machine learning (ML) techniques [13], [14], [15].

However, to develop high-performance ML models for nanotoxicity prediction, it is essential to collect datasets that are high-quality, complete, balanced, and extensive, comprising both physicochemical and toxicological data on NMs. Additionally, effective data preprocessing and model tuning are essential for optimizing these models. In response to these challenges, research efforts have focused on establishing comprehensive guidelines for ensuring data quality [16], [17], [18], and several studies have drawn upon these guidelines to assess data quality and develop robust prediction models. Moreover, many researchers have implemented various data preprocessing techniques, such as filling gaps in missing information and addressing class imbalance [19], [20], [21]. They have also utilized automated machine learning (AutoML) platforms to streamline the model optimization process, reducing the need for manual tuning [3], [22]. Despite these efforts, a challenge remains in one key area. Manual data extraction of high-quality and comprehensive data from vast amounts of literature is extremely time-consuming. This challenge has propelled the adoption of AI-driven tools for data extraction, particularly large language models (LLMs).

LLMs have significantly advanced the field of natural language processing (NLP) by excelling in handling conversational prompts and generating contextually appropriate and coherent responses. With their ability to interpret and create human-like text, these models are invaluable for applications like chatbots, automated text generation, and advanced language comprehension tasks. Recent advancements in LLMs have demonstrated their potential for extracting information, such as clinical data, from reports through prompt engineering, which optimizes input queries to achieve better output [23], [24], [25]. Despite this progress, the application of LLMs for extracting nanotoxicity data has not yet been widely explored. At the same time, numerous efforts have been made to optimize the use of LLMs, one of which is the development of LangChain. LangChain is a framework that helps developers easily create applications using LLMs by integrating them with external data sources and other systems. It extends LLM capabilities by supporting tasks such as text embedding, retrieval, and application programming interface (API) interactions, making it a versatile tool for building complex, real-world applications [26], [27]. It also enables researchers to automate and streamline data extraction, significantly improving efficiency and accuracy. The integration of LLMs with LangChain offers a promising solution to overcome the limitations of manual extraction in nanotoxicity research.

In this study, we developed an AI-driven pipeline to automate the extraction of nanotoxicity data from research papers, ultimately facilitating the development of prediction models. By using LLMs, we aimed to efficiently extract and organize the physicochemical and toxicological properties of NMs into a structured dataset. The pipeline consists of three key steps: (1) Data preparation, which involved gathering publicly available research papers from web databases, (2) Data extraction using LLMs, where the LangChain framework and text embedding techniques were employed, where well-known LLMs such as ChatGPT, Claude, and Gemini were used to compare their effectiveness in extracting relevant nanotoxicity data, and (3) Model development, where the extracted data was used to develop prediction models on AutoML platforms, including Google Vertex AI, Microsoft Azure, Amazon SageMaker, and Dataiku, to assess their reliability and performance. This study highlights the important role of AI in nanotoxicology research, emphasizing its ability to automate the data extraction process. By enabling more efficient development of prediction models, AI has the potential to significantly accelerate advancements in nanotoxicology, leading to a deeper understanding of the toxicological effects of NMs and the development of safer nanotechnology applications.

## 2 Materials and methods

### 2.1 Workflow

**Fig. 1:**
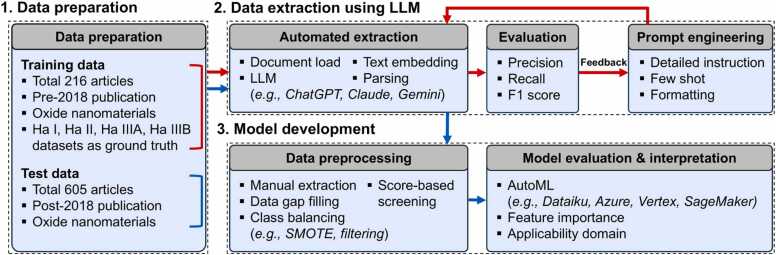
Overall workflow of (1) data preparation, (2) data extraction using LLMs, and (3) model development. The training data undergoes automated extraction, evaluation, and prompt engineering to refine the prompts (red line). The test data is automatically extracted using the selected LLM and prompts, followed by data processing and model development through AutoML (blue line).

The present study followed the workflow illustrated in Fig. 1. The training data were first used to select the appropriate LLM, followed by prompt refinement through an automated data extraction pipeline, where manually curated datasets served as the ground truth for evaluation. Following data extraction, the outputs were evaluated, and feedback was incorporated to refine the prompts. Once the LLM selection and prompt optimization were finalized, data extraction was applied to the test data. Data not captured through automated extraction was manually input. Following data extraction, preprocessing steps, including PChem score-based filtering, gap filling [19], [20], and handling class imbalance [21], [28], were performed. Finally, prediction models were developed using various AutoML platforms, and their performance was evaluated based on metrics such as applicability domain and feature importance.

### 2.2 Data preparation

For downstream analysis, two independent datasets were established: training and test data. A total of 216 research articles related to the toxicity of oxide nanomaterials were sourced from S2Nano. These articles served as training data for optimizing prompts and selecting the LLM with the best performance in data extraction. In a previous study by Ha et al., manually curated datasets were constructed using these 216 research articles [19]. Specifically, the Ha I dataset comprises 6842 toxicity screening data entries for 26 different metal oxide nanoparticles (NPs). Additional datasets—Ha II, Ha IIIA, and Ha IIIB—were derived from Ha I through data gap filling and PChem score-based filtering. These manually curated datasets served as ground truth for evaluating the accuracy of the results extracted by the LLMs. The test data were collected from the PubMed and Web of Science databases, initially yielding 1706 documents related to the toxicity of metal oxide NPs. However, many of these articles lacked the specific attributes required for our analysis. After careful selection, the test data were narrowed down to 605 documents that met the necessary criteria.

### 2.3 Data extraction using LLM

#### 2.3.1 Automated extraction

To efficiently extract data from a large volume of research articles, this study utilized APIs for batch processing and applied text embedding techniques. This approach not only eliminated the need for manual interactions with web-based LLM interfaces but also ensured that irrelevant content was filtered out, allowing the models to focus solely on extracting relevant information. LangChain served as the core framework, integrating multiple APIs: Zotero, an external database, a text embedding model (text-embedding-3-large, Open AI), and LLMs. The process began by retrieving the text and metadata from PDFs and splitting the text into chunks of 300 tokens with an overlap of 50. These chunks were embedded as vectors and stored. The system then retrieved the top 10 nearest vectors to the user's query using the Euclidean distance metric in FAISS. These retrieved chunks, along with the prompt, were input into the LLM, which then generated the output. To correct errors and reformat the model output, LangChain's output parsers, such as the ‘CommaSeparatedListParser’ and ‘OutputFixingParser,’ were employed. Finally, the parsed data was organized into a tabulated format using Python, ensuring a clear and structured dataset for further analysis. To evaluate the effectiveness of various LLMs in extracting nanotoxicity data, three prominent models were tested —ChatGPT 4o, Gemini 1.5 Pro, and Claude 3.5 Sonnet—and their performance in data extraction was compared. By running all models with their default settings (e.g., temperature), we ensured that any differences in extraction performance were solely due to inherent model characteristics rather than parameter adjustments.

#### 2.3.2 A structured framework for extracting nanotoxicity data

**Fig. 2:**
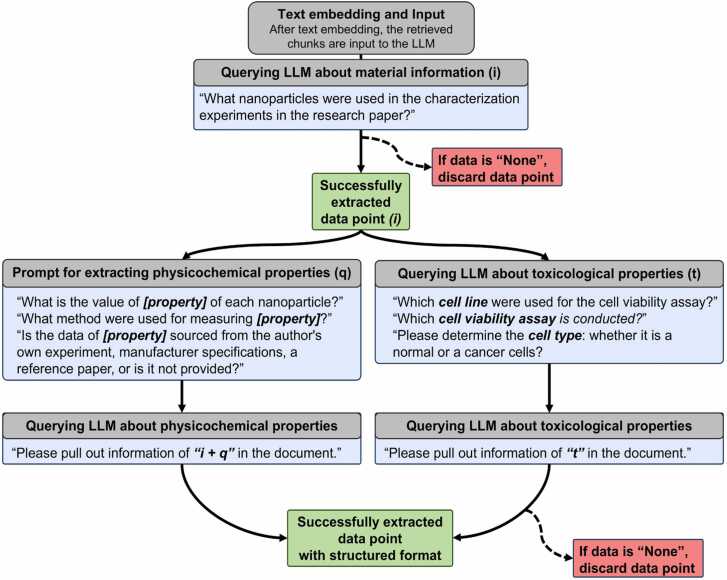
A flowchart illustrating the systematic querying of material information, physicochemical (PChem) properties, and toxicological (Tox) properties. The process begins with querying material information, followed by questions on PChem and Tox properties. For PChem properties, the output from the material information query (i) is combined with specific questions on PChem properties (q) and provided as input to the LLM. Missing data points, where material information or Tox properties are labeled as "None," are used for evaluating extraction performance but are excluded from the final datasets.

When curating research papers for LLMs to process and extract information, it is important to consider the diverse properties related to the cytotoxicity of nanoparticles, such as material types and their physicochemical and toxicological characteristics. Additionally, the interconnections among these properties must be considered. In cases where multiple nanoparticles are used, the material and physicochemical properties of each nanoparticle must be appropriately linked. This requirement serves as the foundation for the data extraction structure shown in Fig. 2. After text embedding, the retrieved chunks were input into the LLM. The process begins with extracting material information (e.g., material type). Subsequently, data related to physicochemical properties (e.g., core size, hydrodynamic size, surface charge, surface area, measurement methods for each property, and data source), and toxicological properties (e.g., cell viability assay, cell line, cell type, cell organ, and cell species) are extracted. For physicochemical properties, the output from the material information query (i) is combined with the physicochemical properties query (q) and provided as input to the LLM. This structured querying approach enables the accurate extraction of physicochemical characteristics specific to each nanoparticle, particularly in research papers involving multiple nanoparticles. In nanotoxicity research papers, material information and toxicological properties are typically present, while physicochemical properties may occasionally be missing. Data points where material information or toxicological properties were labeled as “None” were used for evaluating extraction performance but were excluded from datasets intended for modeling.

On the other hand, cell viability values in toxicological data are key endpoints when developing toxicity prediction models, with dose and exposure time also being significant attributes. However, these values were not considered during the data extraction phase with LLMs. Most research papers present dosage, exposure time, and corresponding cell viability values as XY plots in figures rather than as text. The current LLM version has limitations in extracting specific Y-axis values corresponding to X-axis coordinates from such plots. Consequently, cell viability, dosage, and exposure time values were extracted manually. The types of data extracted automatically and those extracted manually are summarized in Fig. S1.

#### 2.3.3 Prompt engineering

The performance of LLM responses can vary significantly depending on how queries are structured. Refining these queries, known as prompt engineering, is essential for accurately extracting material information, as well as the physicochemical and toxicological properties of NMs. However, the lack of standardization in representing these characteristics makes it challenging to create a prompt that applies to all research papers. To address this, we developed prompt engineering strategies that focus on creating general and comprehensive prompts rather than overly specific ones.

Fig. S2 illustrates the implementation of a prompt template, a core feature provided by LangChain. We assigned specific roles and tasks to the LLM, an approach known as role-prompting, which is one of the fundamental methods in prompt engineering [29]. This technique is particularly effective in guiding the model’s responses and ensuring alignment with the desired output. Additionally, response configuration was used to set the tone of the model's responses.

Prompt engineering was applied to data extraction. As shown in the flowchart in Fig. 2, material information was the first to be extracted. The focus was on ensuring that when multiple nanoparticles were used, they were extracted in a structured format without omissions. Additionally, even if the same type of nanoparticle was referred to by different names, it needed to be identified and extracted separately. To achieve this, we employed detailed instructions and the few-shot prompting technique. Few-shot prompting involves providing several examples of questions and answers to help the model recognize the structure [29], such as ‘Material name (Material type),’ and guide the output accordingly.

**Fig. 3:**
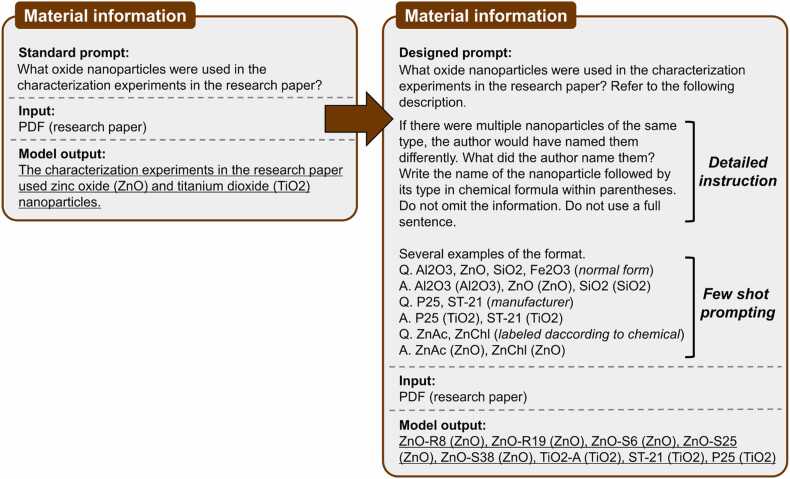
Prompt engineering from a standard prompt (left) to a carefully designed prompt (right) for extracting material information. The refined prompt guides the LLM in adjusting the output format by providing detailed instructions and employing few-shot prompting, ensuring a consistent and structured output format.

As shown in Fig. 3, when the model was queried without additional guidance, the responses were typically presented in an unstructured narrative format, often omitting some of the nanoparticles used. However, providing detailed instructions along with examples of the desired output format resulted in more structured and comprehensive results.

**Fig. 4:**
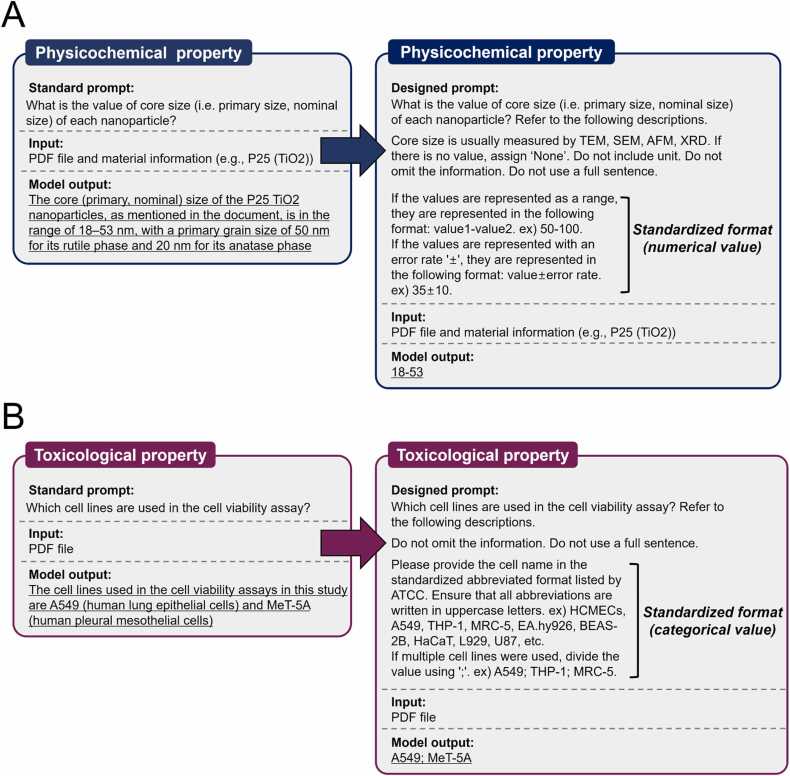
Prompt engineering from a standard prompt (left) to a carefully designed prompt (right) for extracting physicochemical (A) and toxicological (B) properties. For physicochemical properties (A), the prompt standardizes numerical values, including ranges and error margins, while for toxicological properties (B), it enforces standardized categorical formats, such as abbreviations and delimiters, to enhance accuracy and usability in downstream analysis.

Finally, data extraction for PChem and Tox properties was performed. Given that the data includes both numerical and categorical values, extracting it in a standardized format is essential for efficient data preprocessing in subsequent machine learning modeling. To achieve this, we incorporated detailed instructions in the prompt, specifying standardized formats such as number notation, capitalization, abbreviation handling, and the use of semicolons to separate multiple values, as shown in Figs. 4A and 4B. It was observed that using prompts with more detailed and consistent formatting, rather than simpler questions, led to a notable improvement in the uniformity of formatting and reproducibility of the LLM's responses.

We also addressed the challenge of minimizing hallucination, a common issue with LLMs where the models may generate fabricated or misleading content. To mitigate this, we carefully structured prompts to reduce ambiguity and lower the likelihood of hallucination. As noted in other studies [30], [31], well-designed prompts and clear contextual guidance can significantly reduce hallucination occurrences, ensuring the extraction of accurate and relevant data. For example, we included instructions such as *'If there is no value, assign ‘None’.’ a*nd *‘Do not use a full sentence.'* These directives helped refine responses and improved data reliability.

#### 2.3.4 Evaluation of data extraction

The manually curated datasets from the training data were used as the ground truth and directly compared to the data extracted by LLMs for evaluation. Each data point was categorized into one of three labels: true positive (TP; correct assignment), false positive (FP; incorrect assignment), or false negative (FN; failure to extract), as shown in Table 1. In this study, true negatives (TN) were not defined. Based on this classification, Precision_D.E._, Recall_D.E._, and F1_D.E._ (where *‘*_*D.E.*_*’* denotes Data Extraction) were calculated.

**Table 1:** Overview of the three labels used in data extraction assessment. Note that TP, FP, and FN represent true positive, false positive, and false negative, respectively.

Labels	Description
TP	Correct assignment of PChem/Tox attributes by LLM
FP	Incorrect assignment of PChem/Tox attributes by LLM
FN	Failure of LLM to extract PChem/Tox attributes

### 2.4 Data preprocessing

Previous studies have shown that model performance improves with the size and quality of the dataset [22]. Based on this, the automatically extracted dataset was processed using the same data preprocessing approach. Missing PChem data were filled using values from source NPs with similar properties, while commercial materials (e.g., Aeroxide P25) were supplemented with manufacturer-provided data for identical products. Although manually processed, AI-based data extraction categorized the sources of each PChem data point. Specific surface area (SSA) and core size (d) were estimated interchangeably using Eq. (1):

(1) $$\mathrm{SSA}\;=\;\frac{6}{d\;\times \;\rho }$$

Where ρ is the NP density. Missing QM properties were replaced with data from Zhang et al., Gajewicz et al., and Liu et al. [32], [33], [34]. Finally, the dataset was further refined by filtering the top 20 % based on the PChem score to ensure high-quality data selection [19]. To address class imbalance, we applied the Synthetic Minority Over-sampling Technique (SMOTE), which generates synthetic examples to enhance the representation of the minority class and balance the dataset. In particular, SMOTE was applied to the toxicity column. For the material type column, which contains multiple distinct values rather than binary categories like toxicity, class balancing was achieved by filtering and selecting the top seven materials with the highest frequencies.

### 2.5 AutoML development

We developed a prediction model for nanotoxicity using a large dataset of nanotoxicity data. AutoML platforms were used to construct models that predict the cellular toxicity (toxic or nontoxic) of oxide NMs. The model’s predictive endpoint is cell viability (%), determined using cell viability assays. NMs that result in cell viability below 50 % are labeled as "toxic," while those maintaining viability above 50 % are labeled "nontoxic." The criteria for toxic/nontoxic classification can vary depending on experimental conditions (e.g., dose, NM types, and cell types). However, to ensure fair and robust comparisons, we defined the endpoint using the above criteria in accordance with the OECD (Organization for Economic Co-operation and Development) principle of (Q)SAR (quantitative structure-activity relationship) validation, which was also applied in previous studies [35]. We utilized AutoML from Google Vertex AI, Microsoft Azure, Amazon SageMaker, and Dataiku. By comparing performance metrics across these platforms, we aimed to ensure the robustness and reliability of the model. The training and test sets were split using a 6:4 ratio, and hyperparameters were optimized based on the F1_N.P._ (where *‘*_*N.P.*_*’* denotes nanotoxicity prediction). Additionally, k-fold cross-validation (k = 5) was employed to prevent overfitting. The results were reviewed in the model panel of each platform, where we compared performance metrics (e.g., F1_N.P._, Accuracy_N.P._, Precision_N.P._, and Recall_N.P._) derived from confusion matrices.

### 2.6 Model interpretation: Feature importance and applicability domain

In this study, to assess the significance of each feature in the prediction models, we employed the SHAP (Shapley Additive Explanations) method, provided by the AutoML platforms. The applicability domains of the developed models were analyzed using the Euclidean distance approach. A cutoff value, D_c_, defining a distance threshold, was calculated using Eq. (2)

(2) $$D_{c}\;=\;\overset{¯}{D}\;+\;Z\;\times \;s$$

where D is the average distance, s is the standard deviation of the distances from each data sample to other data samples in the training set, and Z is a parameter used to adjust the confidence level. This distance threshold determined whether a new data sample could be reliably predicted by the model. If the distance between the new sample and the training data was smaller than the threshold, the sample was considered sufficiently similar, allowing for predictions with a certain level of confidence. A confidence level of 95 % was chosen for this analysis, corresponding to a one-tailed test with Z = 1.645.

## 3 Results and discussion

### 3.1 Evaluation of data extraction performance

The goal of this study was to develop an automated pipeline for the efficient extraction of nanotoxicity data. To achieve this, we utilized LangChain, which enables retrieval-augmented generation (RAG), allowing the model to directly retrieve information from research papers and generate data-driven responses [36]. LangChain not only connects data sources but also facilitates management by chaining multiple external models, allowing step-by-step responses to user requests [26], [27]. Additionally, its use of text embedding and similarity search to deliver only relevant chunks to the LLM made it highly suitable for our study, which required handling large datasets and extracting multiple parameters [37], [38].

**Fig. 5:**
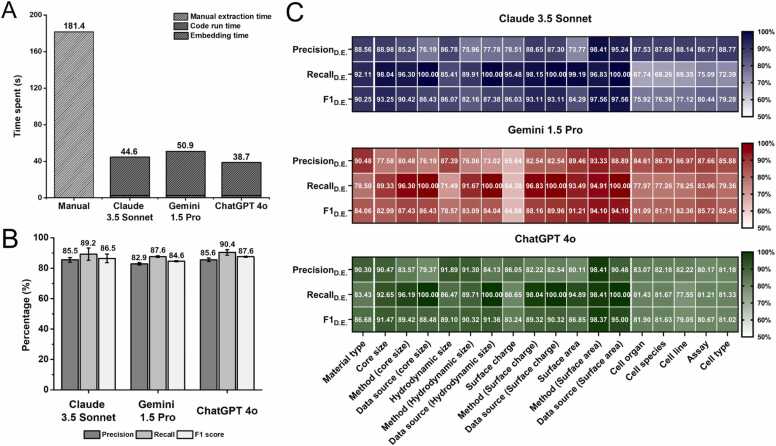
Evaluation of data extraction performance. (A) Comparison of time spent on data extraction per paper using manual extraction versus automated extraction by each LLM. The total time for each method is further broken down into manual extraction time, code run time and embedding time. (B) Average Precision_D.E._, Recall_D.E._, and F1_D.E._ for each LLM, with standard deviations displayed as error bars. (C) Heatmaps showing the percentage of Precision_D.E._, Recall_D.E._, and F1_D.E._ for specific nanotoxicity attributes across each LLM, with color gradients representing performance levels (50 %–100 %).

After building the automated data extraction pipeline, we evaluated its accuracy and efficiency compared to manual extraction methods. A key advantage of LangChain is its flexibility, which allows seamless switching between different LLM models with minimal code modifications. In our evaluation, we employed three prominent LLMs and first compared the time required for data extraction across these models and manual methods using training data. Fig. 5A illustrates the time taken per paper for each method. We found that the automated data extraction method was significantly more time-efficient than manual extraction. On average, manual extraction took 181.4 seconds per paper, approximately 4.11 times longer than using LLMs. Next, we compared the time required for each LLM. As described in the methods section, we used the same text embedding model for automated data extraction, which took an average of 2.6 seconds per paper to process training data. However, response times varied across LLMs after the text embedding process. The time required for LLMs to process input queries and generate outputs averaged 42.0 seconds for Claude 3.5 Sonnet, 48.3 seconds for Gemini 1.5 Pro, and 36.1 seconds for ChatGPT 4o. Including embedding time, the total extraction times were 44.6, 50.9, and 38.7 seconds, respectively, with ChatGPT 4o emerging as the fastest model.

After evaluating time-efficiency, we assessed the accuracy of extraction performance across each LLM in three parts. Given the interconnected nature of data in nanotoxicity papers, we first assessed extraction accuracy for the 'Material type'. Next, physicochemical attributes (e.g., core size, hydrodynamic size, surface charge, and surface area) were evaluated only for data entries where the 'Material type' was correctly identified TP. Finally, toxicological attributes (e.g., cell line, cell organ, cell species, cell type, and cell viability assay), which can have multiple values, were examined. This segmentation was implemented to prevent distortion that can occur when splitting rows for multiple data points, which can lead to excessive duplication of single data points such as physicochemical properties. Fig. 5B summarizes the overall Precision_D.E._, Recall_D.E._ and F1_D.E._ for physicochemical and toxicological attributes. Across all LLMs, Precision _D.E_ values were consistently lower than Recall_D.E._, indicating a higher number of FP than FN. This suggests that LLMs are more likely to extract incorrect data than to fail in extracting relevant data. Among the extracted attributes, “Surface charge” exhibited the highest hallucination rate, with a Precision_D.E._ of 76.73 ± 8.43. We noticed that this hallucination could be attributed to several reasons. Formatting errors can occur due to the diversity of table formats, such as merged cells, multi-layout structures, and line breaks, as well as issues during the text encoding process. We identified several potential causes for these hallucinations. Formatting-related errors arose due to the diversity of table structures, including merged cells, multi-layout formats, and line breaks, as well as issues related to text encoding. The lack of standardized data formatting contributed to misinterpretations of table headers, while encoding errors led to symbols being misclassified as numerical values. This issue was particularly evident in “Surface charge”, where en dashes and em dashes—commonly used to denote negative charges—were sometimes misinterpreted as numbers, resulting in incorrect extractions. In addition to formatting errors, content-based hallucinations also occurred. A notable example was in the discussion sections of research papers, where results from other studies were frequently cited for comparison. This led to LLMs mistakenly recognizing these citations as relevant chunks, increasing the number of FP entries. Another common error involved core size and hydrodynamic size, which share the same unit (nm) and are often mentioned together. The model sometimes extracted values for one attribute but misclassified them as the other, indicating more FP data entries. When comparing the overall F1_D.E._ of each LLM, Claude 3.5 Sonnet achieved a score of 86.5, Gemini 1.5 Pro scored 84.6, and ChatGPT 4o had a slightly higher score of 87.6. As a result, we confirmed that among the three LLMs, ChatGPT 4o demonstrated the best performance.

Following the overall assessment, each attribute was individually evaluated to examine its specific characteristics. Fig. S3 presents the distribution ratios of TP, FP, and FN labels for each of the 18 parameters. In the evaluation of ‘method’ and ‘data source,’ these are not values used as features in the prediction model but are instead factors considered in the calculation of the PChem score (Table S1). In other words, filtering is performed based on the PChem score, and these attributes indicate how accurately that filtering functions. We observed that the attributes ‘method’ and ‘data source’ exhibited a higher TP rate for Claude 3.5 Sonnet, Gemini 1.5 Pro, and ChatGPT 4o (83.93 ± 8.49, 79.76 ± 6.19, and 84.72 ± 5.55, respectively) than other attributes (70.90 ± 7.62, 69.14 ± 8.89, and 72.58 ± 6.12, respectively). We believe that instructing the model to choose from predefined answers such as *‘Just answer four types: Experiment, Manufacturer, Reference, Not specified’* or specifying commonly used measurement methods increase the likelihood of extracting TP entries (Table S2).

Next, Precision_D.E._, Recall_D.E._, and F1_D.E._were calculated for each attribute based on the ratio of data entry labels, and these results were visualized through heatmaps, as shown in Fig. 5C. The heatmaps revealed notable differences in the Recall_D.E._ of physicochemical and toxicological attributes across the LLMs. For Claude 3.5 Sonnet, the Recall_D.E._ value for physicochemical attributes was 94.53 ± 5.43, while for toxicological attributes, it was 70.56 ± 2.77. For Gemini 1.5 Pro, the Recall_D.E._ value for physicochemical attributes was 79.67 ± 12.09, while for toxicological attributes, it was 79.36 ± 2.40. For ChatGPT 4o, the Recall _D.E_ value was 88.66 ± 3.42 for physicochemical attributes and 80.64 ± 1.55 for toxicological attributes. These results show that physicochemical properties, which consist of single data points, consistently exhibit higher Recall_D.E__._ values compared to toxicological properties, which can involve multiple data points. There are two main reasons for this discrepancy. First, errors can arise during the text splitting process. When splitting text into chunks of 300 tokens, relevant content may be omitted. Second, ChatGPT is known to extract only a subset of multiple values, which could contribute to this issue (often referred to as 'laziness'). Similar tendencies were observed in other LLMs as well. Even when the prompt explicitly included the instruction "*Please tell me all the (parameters), without omission*" the models frequently failed to extract all values. While OpenAI has acknowledged this limitation and made improvements in ChatGPT 4o and later versions, deficiencies remain, as evidenced in this study.

Overall, among the evaluated LLMs, ChatGPT 4o demonstrated the best performance in terms of time efficiency and accuracy. Its Precision_D.E._ ranged from 79.37 to 98.41 (85.54 ± 5.17), Recall_D.E._ from 77.55 to 100.00 (90.20 ± 8.16), and F1_D.E._ from 79.05 to 98.37 (87.46 ± 5.15). While these metrics were slightly lower than those reported in a similar study using text embedding for MOF synthesis conditions (F1 score of 92.33 ± 3.09) [30], they remain robust given the complexity of nanotoxicity data. Consequently, ChatGPT 4o was selected for downstream analysis, demonstrating its suitability for automating large-scale nanotoxicity data extraction.

### 3.2 Datasets used for model development

Given the extensive range of physicochemical and toxicological properties obtained through our automated data extraction pipeline, our objective was to use these data to curate and predict the toxicity of NMs. We extracted physicochemical properties along with biological properties related to in vitro toxicity tests from the test data. Additionally, using PlotDigitizer, we manually extracted data for dose, exposure time, and cell viability, as these parameters were not directly handled during the automated data extraction process. Moreover, quantum-mechanical properties (e.g., enthalpy of formation (ΔHsf), conduction band (Ec), valence band (Ev), and electronegativity (χMeo)) were incorporated from relevant references [32], [33], [34]. Building on previous studies that demonstrated the benefits of PChem score-based filtering and data gap filling in improving model performance, we employed the same approaches: filtering based on the highest 20 % of PChem scores, followed by data gap filling using manufacturers’ specifications and/or estimations. The resulting dataset, HaHa-Auto, contains 15 descriptors, 2696 rows, and an average PChem score of 4.8 ± 0.1 (Table 2). For comparison, the HaHa-Manual dataset was manually curated from test data with no missing values (FN entries) or errors (FP entries). It contains 3440 rows, with an average PChem score of 4.8 ± 0.1. This dataset served as a reference for evaluating the model performance against the ChatGPT-generated dataset, labeled as HaHa-Auto. Another dataset, Ha IIIB, was also manually curated follows the same data quality criteria as HaHa-Auto. It was designed to compare model performance and applicability domain when ChatGPT is used to generate high-quality datasets of larger size. The Ha IIIB dataset contains 666 rows and has an average PChem score of 4.9 ± 0.1.

**Table 2:** Description of datasets used for modeling.

Material type	Dataset name	Source	No. rows	No. descriptors	PChem Score	Dataset description	Descriptors
Oxide NPs	HaHa-Auto	Test data: 130 articles*	2696	15	4.8 ± 0.1	- Automatically extracted datasets from test data - Data gap filled and filtered 20% highest PChem score from 605 articles	core size, hydrodynamic size, surface charge, surface area, ΔHsf, Ec, Ev, χMeo, cell viability assay, cell line, cell species, cell organ, cell type, exposure time, dose.
	HaHa-Manual	Test data: 130 articles*	3440	15	4.8 ± 0.1	- Manual curated dataset from test data - Data gap filled and filtered 20% highest PChem score from 605 articles	
	Ha IIIB	Training data: 17 articles*	666	15	4.9 ± 0.1	- Manual curated dataset from training data - Data gap filled and filtered 20% highest PChem score from 216 articles	

### 3.3 Model development using AutoML platforms

**Fig. 6:**
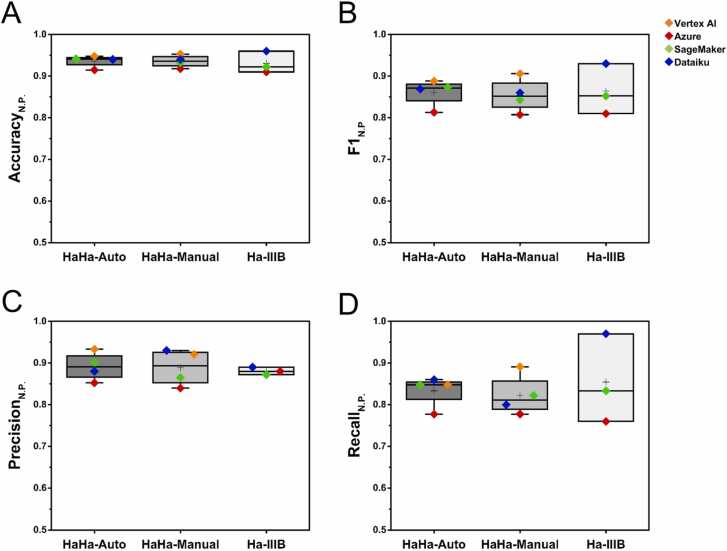
Performance metrics of AutoML models trained on HaHa-Auto, HaHa-Manual, and Ha IIIB datasets. The box plots show (A) Accuracy_N.P._, (B) F1_N.P._, (C) Precision_N.P._, and (D) Recall_N.P_., comparing results across Vertex AI, Azure, SageMaker, and Dataiku. Each colored dot represents the best-performing model on each platform. The black hoizontal line represents the median, while the plus sign indicates the mean value. Note that the Ha IIIB dataset did not meet the required threshold of 1000 rows, necessary for model training on Vertex AI. Therefore, performance metrics for Vertex AI on Ha IIIB are not shown.

**Fig. 7:**
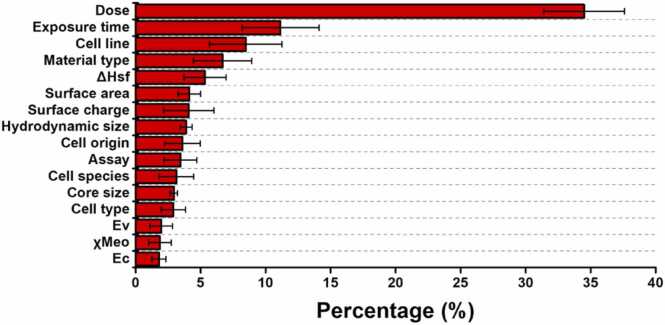
The feature importance of model trained on HaHa-Auto dataset. The chart represents the mean and standard deviation of feature importance across each AutoML platform.

Using our datasets, we developed nanotoxicity prediction models across various AutoML platforms. In our previous research, we observed that AutoML generally outperformed conventional coding-based machine learning in model performance [22]. Additionally, AutoML platforms offer a range of algorithms, enabling users to test various combinations and select the best models based on performance comparisons [39], [40], [41], [42]. As shown in Fig. S4, the Dataiku AutoML platform allows users to select algorithms from a pool to test, making it easier to identify the best-performing model based on key metrics. To minimize the risk of overfitting or bias that could arise from relying on a single platform's algorithms, we employed multiple AutoML platforms. We selected prominent AutoML platforms including Vertex AI, Azure, SageMaker, and Dataiku. Table S3 provides detailed information on the specific algorithms that achieved the best performance, as well as the data preprocessing steps applied in each case. The highest performance metrics from each platform are summarized in Table S4. Based on these results, the performance of the AutoML-based models is illustrated in Fig. 6. The results show that the model performance remains relatively consistent across the datasets. Based on the F1_N.P._, the performance for each dataset was 86.09, 85.41, 86.41 for HaHa-Auto, HaHa-Manual, and Ha IIIB, respectively, with a small standard deviation ranging from 2.85 to 4.97. A similar trend was observed for Accuracy_N.P._, Precision_N.P._, and Recall_N.P._, with minimal standard deviations. These results suggest that using multiple AutoML platforms provides consistent and reliable performance across different datasets. We also analyzed the feature importance, which is essential for understanding how physicochemical and toxicological properties influence model decisions. Fig. 7 presents SHAP-based feature importance, revealing that all AutoML platforms consistently identified similar features—dose, exposure time, cell line and material type—as important. To further enhance model interpretability, Fig. S5 illustrates the partial dependence of individual nanomaterial types, providing deeper insights into how specific features influence predicted cell viability and nanotoxicity.

### 3.4 The reliability and applicability of models trained on ChatGPT 4o-Extracted data

To evaluate the reliability of models trained on ChatGPT-extracted data and explore the advantages of large-scale data extraction, we compared their performance metrics and applicability with models trained on (1) a manually curated dataset, and (2) a smaller dataset. First, we compared the HaHa-Auto and HaHa-Manual datasets. The HaHa-Manual dataset represents the ground truth for test data, whereas the HaHa-Auto dataset may contain missing values or slight discrepancies due to automated extraction. As shown in Fig. 6 and Table S4, no significant differences were observed between models trained on these datasets in terms of Accuracy_N.P._, Precision_N.P._, and Recall_N.P._. However, the F1_N.P._, for the model trained on HaHa-Auto was slightly higher at 86.09 ± 2.85 compared to 85.41 ± 3.55 for HaHa-Manual. While data extraction accuracy remains an important consideration, these results indicate that models built with automatically extracted data perform comparably to those trained on manually curated datasets. This highlights the practicality of automated data extraction pipelines for model development and suggests that further improvements in extraction accuracy could enhance their reliability even more.

**Fig. 8:**
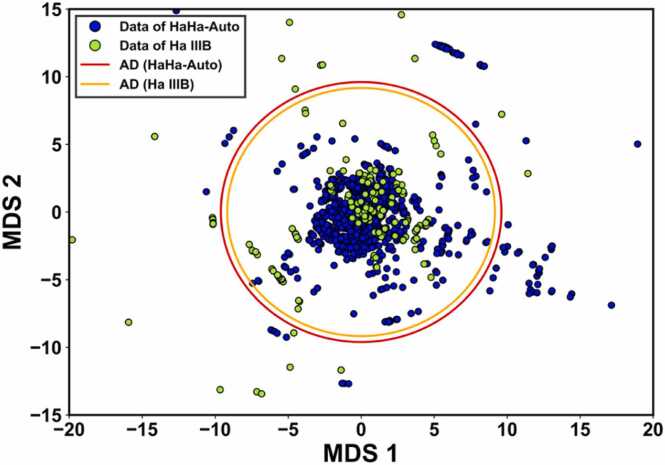
Comparison of the applicability domain (AD) of models trained on HaHa-Auto and Ha IIIB datasets for numerical attributes. The thresholds are determined based on the 95 % confidence level of the Euclidean distance distribution. Only the training data from each dataset is visualized.

Expanding upon the comparison in terms of ground truth, we evaluated the impact of dataset size by comparing models trained on the HaHa-Auto and Ha IIIB datasets. Although both datasets meet the same quality criteria, they differ significantly in size (Table 2): HaHa-Auto contains 2698 rows compared to 666 rows in Ha IIIB. The Ha IIIB dataset was created through manual curation, filtering only data with high PChem scores based on data quality criteria. Therefore, while Ha IIIB is considered a high-quality dataset, it is limited in quantity. To address this limitation, our automated data extraction approach was designed to enable the large-scale production of high-quality data. Furthermore, we compared the models' applicability domain (AD)—the range within which the model can make reliable predictions [43]. We hypothesized that large-scale data production increases the likelihood of obtaining diverse datasets, potentially expanding the model's AD. To define the AD, we employed Euclidean distance, which is simple, intuitive, and independent of the model's algorithm. This makes it particularly suitable for our research, which involves various AutoML algorithms. As shown in Fig. 6 and Table S4, the F1_N.P._, for models trained on the HaHa-Auto dataset was 86.09 ± 2.85, whereas models trained on the Ha IIIB dataset achieved an F1_N.P._ of 86.41 ± 4.97. In addition, improvements were observed across all other performance metrics, suggesting that model performance improves with a larger dataset size. Fig. 8 compares the AD for numerical attributes. The results suggest that even though the HaHa-Auto dataset is larger, the AD for numerical attributes does not significantly expand. This limited expansion is likely due to the limited diversity in the physicochemical properties—such as material composition, particle sizes, and surface charges—extracted from the collected literature. To effectively expand the numerical AD, future studies should include more diverse and extensive research articles for data extraction.

In contrast, Table S5 presents the AD for categories, demonstrating that HaHa-Auto covers a wide range of categories, including material type, assay method, cell species, and cell organ. Although expansions of the numerical AD were limited in this study, the increasing number and diversity of research articles for data extraction in future research could help improve AD for numerical attribute coverage. Despite these potential expansions, maintaining accuracy in automated data extraction remains crucial to ensure the data’s reliability, comparable to manually curated datasets.

Most classification algorithms experience reduced performance on highly imbalanced datasets due difficulties in effectively identifying minority class samples [44], [45]. Although our automated data extraction pipeline successfully extracted a large amount of high-quality data based on the PChem score, it did not address class imbalance in terms of toxicity and material types, as shown in Fig. S6. To address this issue, we applied two data preprocessing techniques. First, SMOTE was applied on the HaHa-Auto dataset to generate synthetic toxic samples. Second, material type balancing was performed by equalizing sample counts through filtering. As a result, the model trained on the SMOTE-processed dataset exhibited improved Recall_N.P._. (93.48 ± 4.92), which is particularly valuable for enhancing sensitivity in detecting toxic samples, an important aspect of risk assessment applications. In contrast, the model trained with material balancing showed a decrease in overall performance. However, the key advantage of this approach is that it prevents the model from becoming biased toward any specific material types, promoting fairness and robustness in prediction outcomes. Therefore, after automatically extracting the data, examining their distribution and applying appropriate preprocessing steps can further improve of our automated data extraction and prediction model.

## 4 Conclusion

In this study, we successfully developed an AI-driven automated pipeline capable of extracting nanotoxicity data from scientific literature, significantly reducing the time and effort required for data collection. Using LLMs, our pipeline efficiently extracted key physicochemical and toxicological properties, producing high-quality datasets for model training. Notably, a comparative analysis identified ChatGPT-4o as the most effective model for this purpose, highlighting its utility in nanotoxicity research. Furthermore, applying these datasets in AutoML platforms enabled the development of prediction models with performance comparable to manually curated methods, while also expanding the applicability domain. This allows researchers to more comprehensively evaluate potential toxicity risks of NMs.

Despite these achievements, certain challenges remain. One limitation is the difficulty in extracting specific quantitative data from precise XY coordinates in graphs, as many studies present essential cell viability data in this format. While LLMs have advanced in text extraction from images, accurately retrieving data points from graphical coordinates remains challenging. Future research could address this through exploring automated methods ranging from OCR-based techniques and advanced computer vision approaches, including object detection and semantic segmentation. By integrating these strategies with multi-modal fusion techniques that combine visual cues and contextual information from axis labels, we aim to significantly enhance the precision and scalability of data extraction, a direction that will be rigorously validated in our future work.

To improve the accessibility and usability of our AI-based nanotoxicity data extraction system, development of an interactive web-based platform would be beneficial to researchers in this field, which will enable researchers to upload their scientific articles, automatically extract key physicochemical and toxicological information, and generate prediction models through integrated AutoML services. However, it is currently challenging due to reliance on paid resources such as GPT API tokens and cloud-based AutoML services from providers like Google, Microsoft, Amazon and Dataiku. As a result, establishing a fully open and cost-free interactive website is difficult.

Nevertheless, as an initial step toward broader accessibility, we have publicly shared our source code on GitHub (https://github.com/yoon-lab/AI-Based-Nanotoxicity-Data-Extraction-and-Prediction-of-Nanotoxicity). Additionally, extracted datasets and prediction models will be available through portals like S2Nano (Safe and Sustainable Nanotechnology, http://s2nano.org/), as well as through consortiums such as INSIGHT (https://insight-project.org/), CompSafeNano (https://compsafenano.eu/) and NSMC (https://safenano.re.kr/). This initiative enhances transparency and facilitates the wider adoption of our work within the nanotoxicology research community.

The AI-driven data extraction pipeline established in this study has significant implications for the future of nanotoxicology research. By enabling efficient data extraction, organization, and prediction modeling, it accelerates the understanding of the toxicological impacts of NMs and facilitates safer applications in nanotechnology. Further advancements in LLMs also open up possibilities for more accurate data extraction, providing researchers with a valuable tool to gain insights without the challenges of manual data processing. These advancements represent a transformative step in nanotoxicity research, potentially enabling the design of safer NMs and enhancing data accessibility.

## CRediT authorship contribution statement

**Ha Eunyong:** Writing – review & editing, Writing – original draft, Visualization, Software, Investigation, Data curation. **Ha Seung Min:** Writing – review & editing, Visualization, Validation, Software, Investigation, Data curation. **Gerelkhuu Zayakhuu:** Writing – review & editing, Validation. **Kim Hyun-Yi:** Software, Data curation. **Yoon Tae Hyun:** Writing – review & editing, Supervision, Methodology, Conceptualization.

## Declaration of Generative AI and AI-assisted technologies in the writing process

During the preparation of this work the authors used generative AI such as ChatGPT, Gemini, Claude, as well as AI-assisted writing tools like DeepL, to aid in translation and sentence rephrasing for improved readability. After using these tools, the authors reviewed and edited the content as needed and take full responsibility for the content of the publication.

## Declaration of Competing Interest

The authors declare that they have no known competing financial interests or personal relationships that could have appeared to influence the work reported in this paper.
