# Supplementary material for: AI-based nanotoxicity data extraction and prediction of nanotoxicity
Source: Comput Struct Biotechnol J. 2025 Apr 3;29:138–48. doi: 10.1016/j.csbj.2025.03.052 (PMC12008667; doi:10.1016/j.csbj.2025.03.052)
Supplement: Supplementary file 1 — Supplementary material [file mmc1.docx]

**AI-Based Nanotoxicity Data Extraction and Prediction of Nanotoxicity**

**Eunyong Ha^a^, Seung Min Ha^a^, Zayakhuu Gerelkhuu^b,c^, Hyun-Yi Kim^d^, and Tae Hyun Yoon^a,b,c,e*^**

^a^Department of Chemistry, Hanyang University, Seoul 04763, Republic of Korea.

^b^Research Institute for Convergence of Basic Science, Hanyang University, Seoul 04763, Republic of Korea.

^c^Institute of Next Generation Material Design, Hanyang University, Seoul 04763, Republic of Korea.

^d^NGeneS Inc., Ansan-si 15495, Republic of Korea.

^e^Yoon Idea Lab. Co. Ltd., Seoul 04763, Republic of Korea.

__________________________________________________________________________

*Corresponding authors. Tel: +82-(0)2-2220-4593

E-mail: [taeyoon@hanyang.ac.kr](mailto:taeyoon@hanyang.ac.kr)

# Supporting information

Fig. S1 Examples of the types of data and extraction results for automated extraction and manual extraction.

**Fig. S2** The prompt template as a feature of LangChain for LLM configuration. The prompt provides necessary context to help the model better understand the task's background and requirements.

Fig. S3 True Positive (TP), False Positive (FP), and False Negative (FN) label count ratios across parameters for each language model. (A) Claude 3.5 Sonnet, (B) Gemini 1.5 Pro, and (C) ChatGPT 4.0. Each bar represents the percentage distribution of TP, FP, and FN labels for each attribute.

Fig. S4 Performance metrics of models built using various algorithms on the Dataiku platform. Each heatmap shows (A) Accuracy_N.P._, (B) F1_N.P._, (C) Precision_N.P._, and (D) Recall_N.P._ for three different datasets: HaHa-Auto, HaHa-Manual, and Ha IIIB. The color gradient represents performance scores, with red indicating higher values and blue indicating lower values. (GBT: gradient boosted trees, LightGBM: light gradient boosted machine, RF: random forest, XGB: XGBoost, DT: decision tree, KNN: k-nearest neighbor, ET: extra trees, LR: logistic regression, SGD: stochastic gradient, LLARS: LASSO-LARS, SLP: single layer perceptron, SVM: support vector machine).

**Fig. S5** Plot shows the dependence of the predicted response on a single feature. These PDPs correspond to the top four features with the highest importance. For the categorical features (A–B), the x-axis displays the partial dependence and distribution, with the y-axis listing the feature categories. For the continuous features (C–D), the x-axis represents the feature values while the y-axis shows the distribution and partial dependence, the change in log-odds relative to the average probability.

Fig. S6 Class balancing of toxicity and material types. (A) Bar graph showing the distribution of toxic and non-toxic data rows in the HaHa-Auto dataset before and after applying the SMOTE algorithm for class balancing. (B) Pie charts depicting the distribution of the top seven material types before and after filtering. The number of data points for each material type was adjusted to match the count of CeO₂, ensuring a balanced dataset for subsequent analysis.

Table. S1 Pchem scoring criteria. TEM: Transmission Electron Microscopy; SEM: Scanning Electron Microscopy; AFM: Atomic Force Microscopy; XRD: X-Ray Diffraction; DLS: Dynamic Light Scattering; NTA: Nanoparticle Tracking Analysis; BET: Brunauer-Emmett-Teller method.

**Table. S2** Summary of prompts not covered in the main text.

Table. S3 Data preprocessing methods and algorithms used in different AutoML platforms.

Table. S4 Comparison of AutoML performance metrics across multiple datasets. Note that the Ha IIIB dataset did not meet the required threshold of 1,000 rows, which is necessary for model training on Vertex AI. Therefore, the metrics for Vertex AI on Ha IIIB are not shown.

Table. S5 Applicability domain analysis for categorical attributes. The number in parentheses represents the number of components included in each dataset.

# Figures


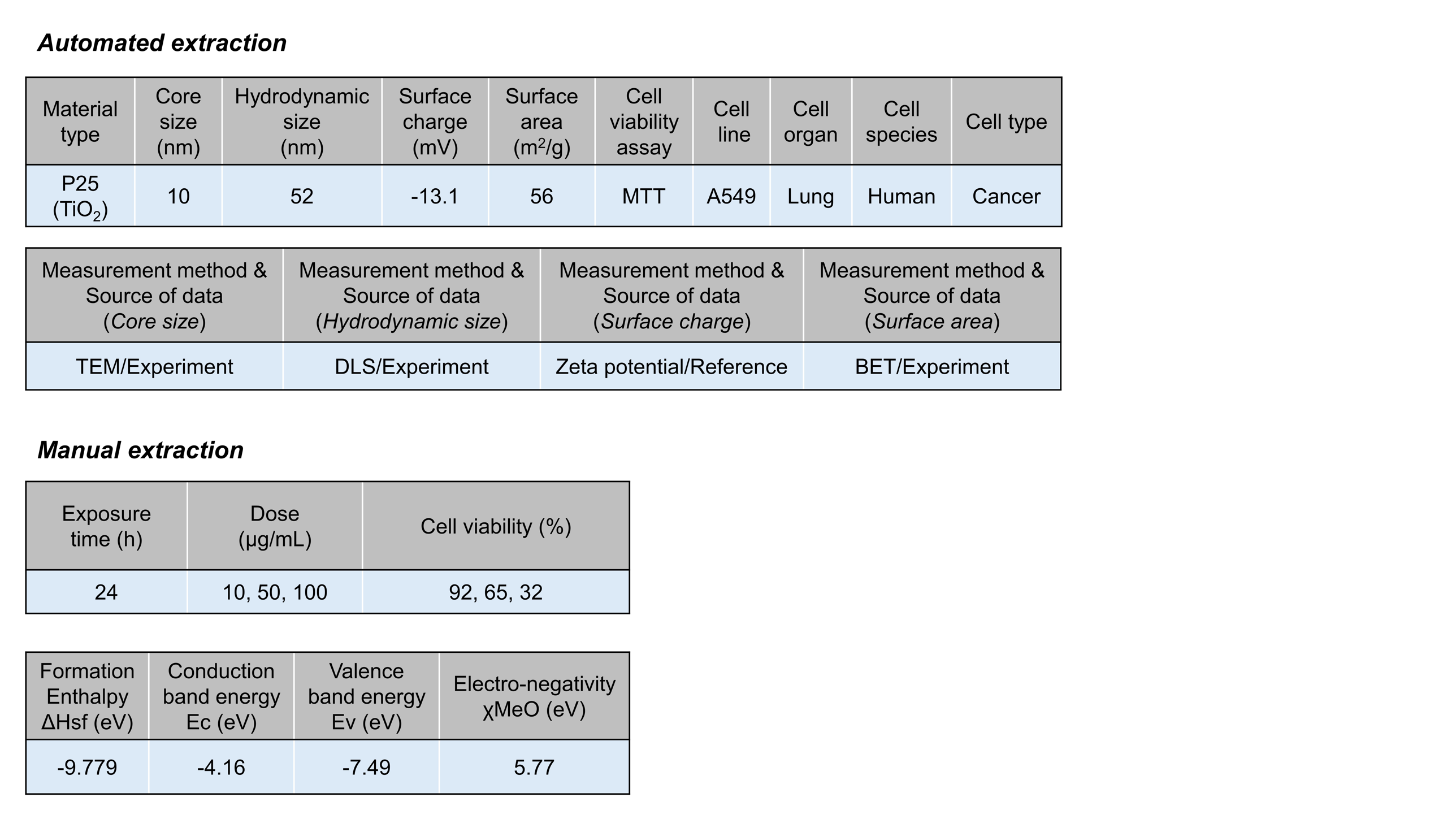


**Fig. S1.** Examples of the types of data and extraction results for automated extraction and manual extraction.


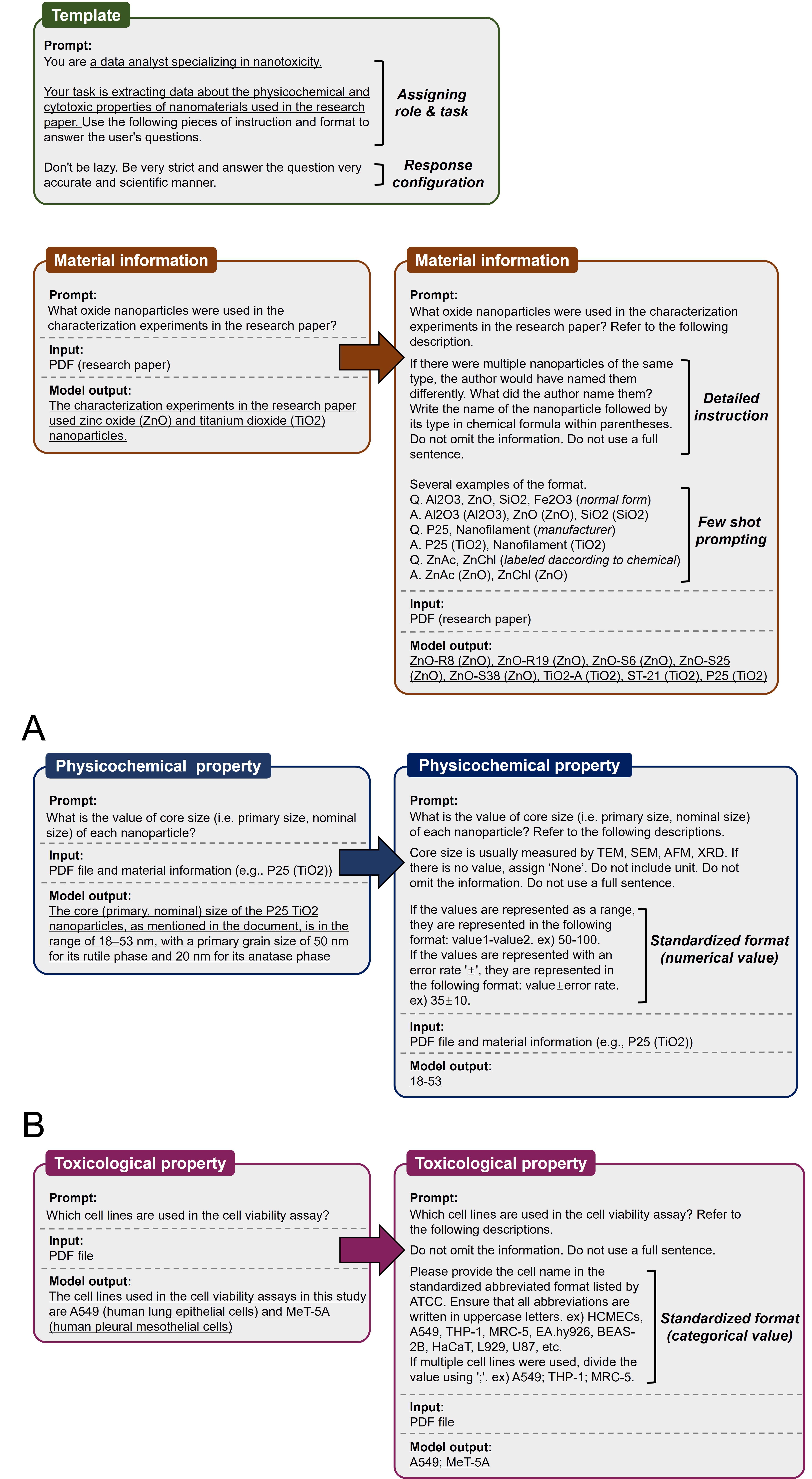


**Fig. S2.** The prompt template as a feature of LangChain for LLM configuration. The prompt provides necessary context to help the model better understand the task's background and requirements.


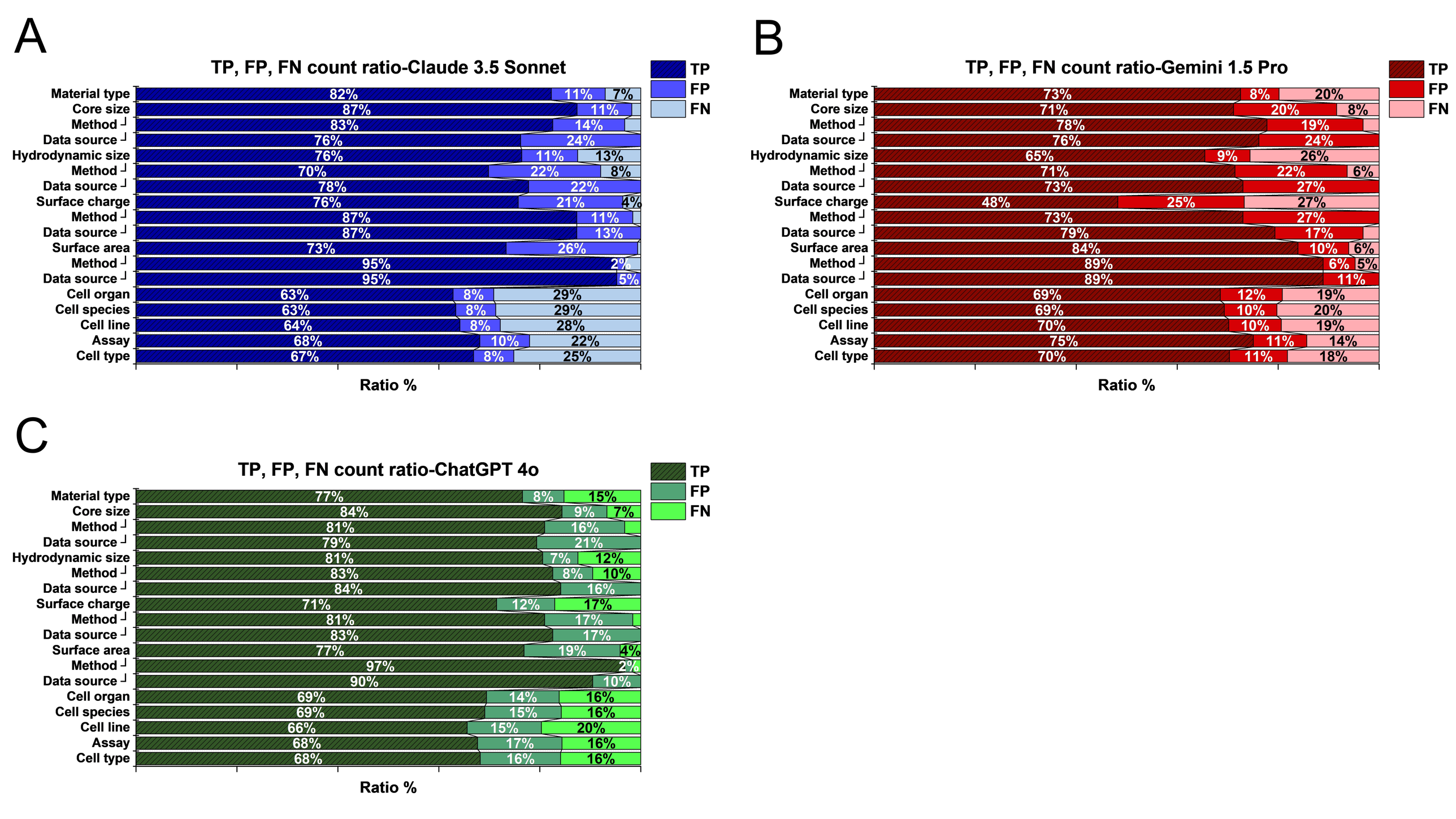


**Fig. S3.** True Positive (TP), False Positive (FP), and False Negative (FN) label count ratios. (A) Claude 3.5 Sonnet, (B) Gemini 1.5 Pro, and (C) ChatGPT 4.0. Each bar represents the percentage distribution of TP, FP, and FN labels for each attribute.


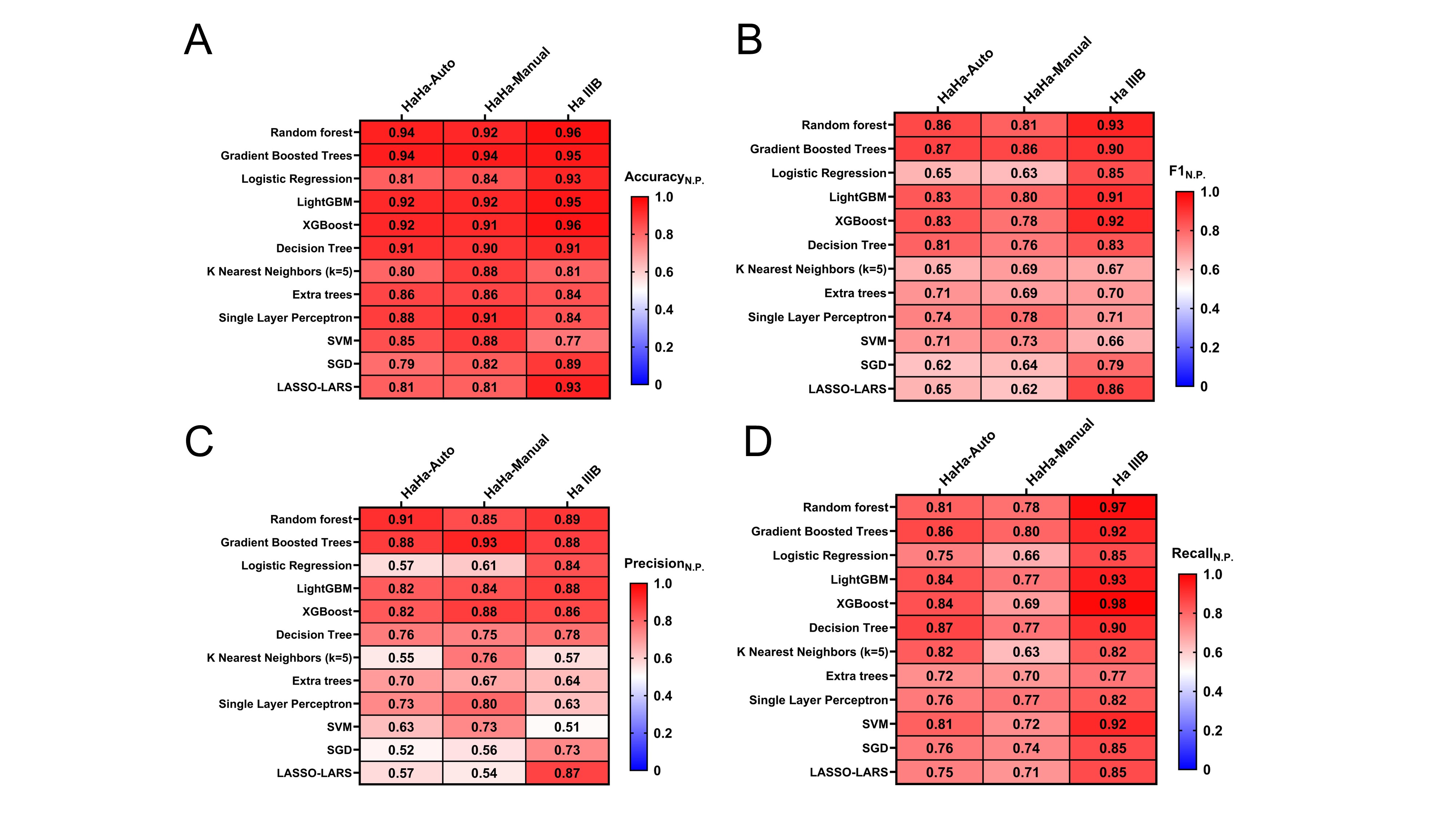


**Fig. S4.** Performance metrics of models built using various algorithms on the Dataiku platform. Each heatmap shows (A) Accuracy_N.P._, (B) F1_N.P._, (C) Precision_N.P._, and (D) Recall_N.P._ for three different datasets: HaHa-Auto, HaHa-Manual, and Ha IIIB. The color gradient represents performance scores, with red indicating higher values and blue indicating lower values. (GBT: gradient boosted trees, LightGBM: light gradient boosted machine, RF: random forest, XGB: XGBoost, DT: decision tree, KNN: k-nearest neighbor, ET: extra trees, LR: logistic regression, SGD: stochastic gradient, LLARS: LASSO-LARS, SLP: single layer perceptron, SVM: support vector machine).


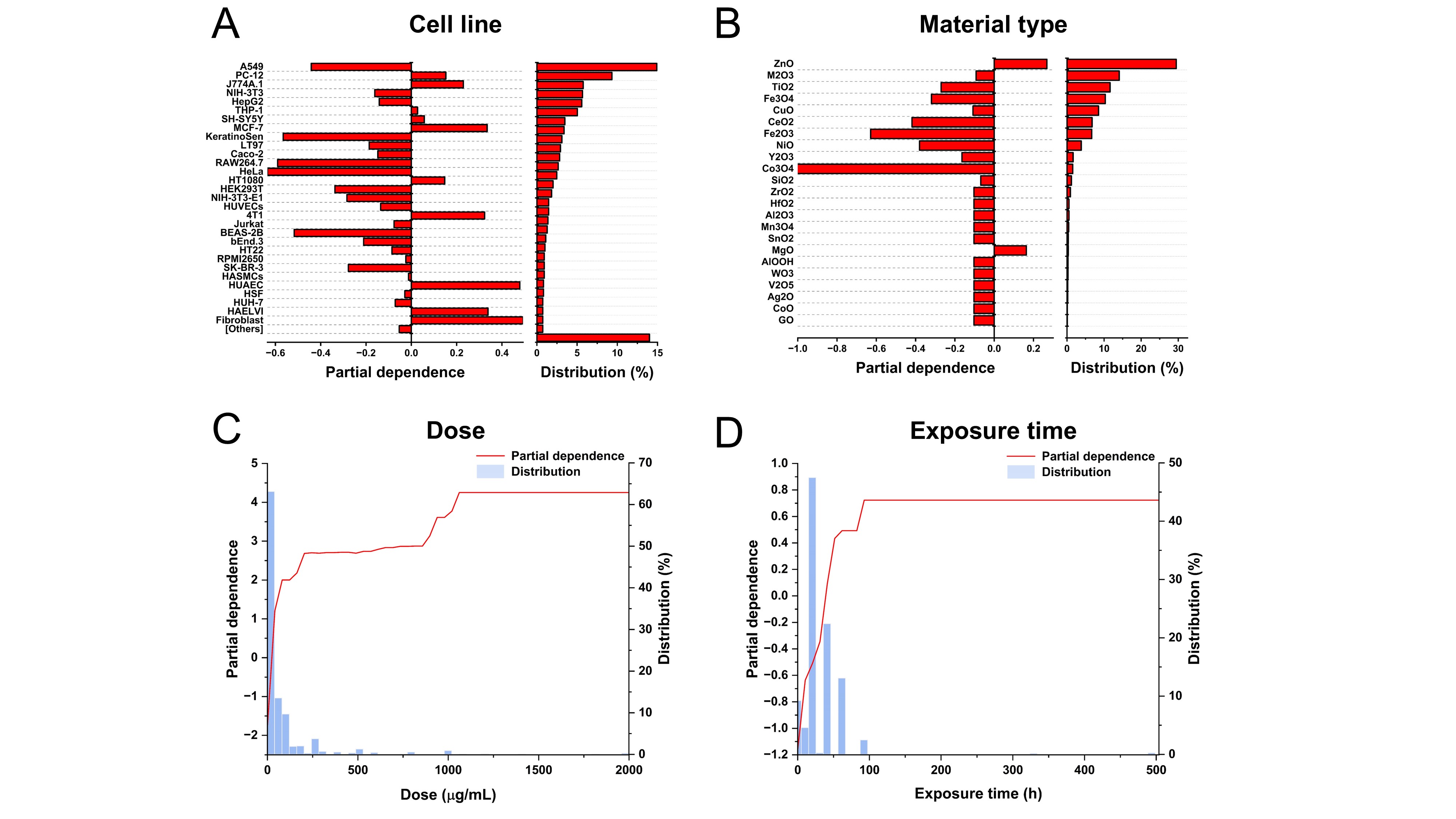


**Fig. S5.** Plot shows the dependence of the predicted response on a single feature, based on results from Dataiku platform. These PDPs correspond to the top four features with the highest importance. For the categorical features (A–B), the x-axis displays the partial dependence and distribution, with the y-axis listing the feature categories. For the continuous features (C–D), the x-axis represents the feature values while the y-axis shows the distribution and partial dependence, the change in log-odds relative to the average probability.


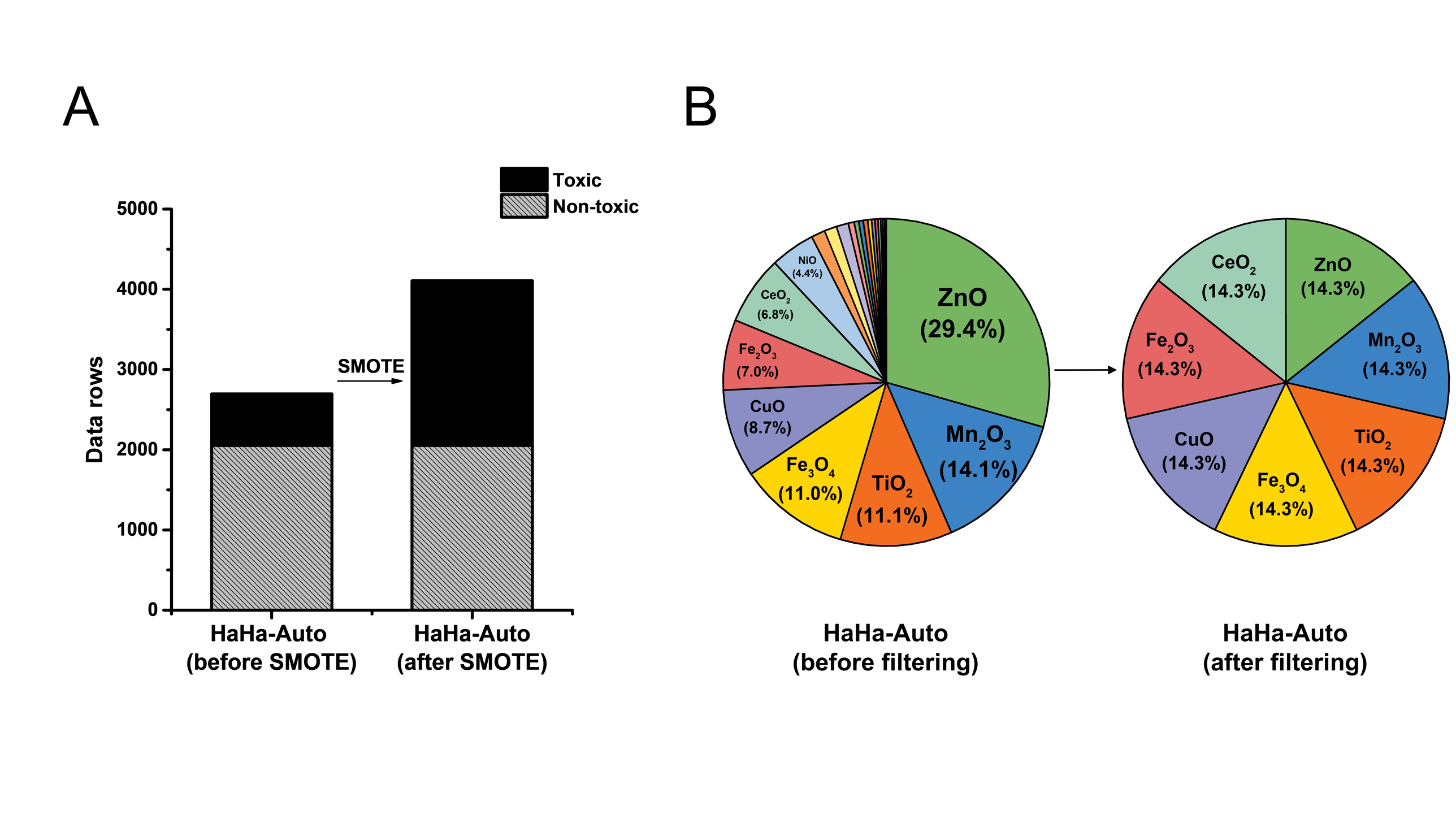


**Fig. S6.** Class balancing of toxicity and material types. (A) Bar graph showing the distribution of toxic and non-toxic data rows in the HaHa-Auto dataset before and after applying the SMOTE algorithm for class balancing. (B) Pie charts depicting the distribution of the top seven material types before and after filtering. The number of data points for each material type was adjusted to match the count of CeO₂, ensuring a balanced dataset for subsequent analysis.

# Tables

**Table S1.** Pchem scoring criteria. TEM: Transmission Electron Microscopy; SEM: Scanning Electron Microscopy; AFM: Atomic Force Microscopy; XRD: X-Ray Diffraction; DLS: Dynamic Light Scattering; NTA: Nanoparticle Tracking Analysis; BET: Brunauer-Emmett-Teller method.

| Attribute | Criteria | Description | Score |
| --- | --- | --- | --- |
| Core size | Data source | -Experimentally measured by the authors | 3 |
|  |  | -Adapted from manufacturers’ specification | 2 |
|  |  | -Adapted from other reference using same nanomaterials and experimental conditions | 1 |
|  |  | -No data | 0 |
|  | Data method | -TEM | 2 |
|  |  | -Estimated from specific surface area – Other methods (e.g., SEM/AFM) | 1 |
|  |  | -No data | 0 |
| Hydrodynamic size | Data source | -Experimentally measured by the authors | 3 |
|  |  | -Adapted from manufacturers’ specification | 2 |
|  |  | -Adapted from other reference using same nanomaterials and experimental conditions | 1 |
|  |  | -No data | 0 |
|  | Data method | -DLS/NTA | 2 |
|  |  | -Other methods | 1 |
|  |  | -No data | 0 |
| Surface charge | Data source | -Experimentally measured by the authors | 3 |
|  |  | -Adapted from manufacturers’ specification | 2 |
|  |  | -Adapted from other reference using same nanomaterials and experimental conditions | 1 |
|  |  | -No data | 0 |
|  | Data method | -Zeta potential | 2 |
|  |  | -Other methods | 1 |
|  |  | -No data | 0 |
| Surface area | Data source | -Experimentally measured by the authors | 3 |
|  |  | -Adapted from manufacturers’ specification | 2 |
|  |  | -Adapted from other reference using same nanomaterials and experimental conditions | 1 |
|  |  | -No data | 0 |
|  | Data method | -BET | 2 |
|  |  | -Estimated from core size – Other methods | 1 |
|  |  | -No data | 0 |

**Table S2.** Summary of prompts not covered in the main text.

| **Attributes** | **Designed prompts** | **Outputs** |
| --- | --- | --- |
| Surface area | What is the value of surface area of each nanoparticle? Refer to following descriptions. 1. Surface area usally measured by BET. Do not include unit. Do not omit the information. Do not use a full sentence. 2. If the values are represented as a range, they are represented in the following format: value1-value2. ex) 50-100. 6. If the values are represented with an error rate '±', they are represented in the following format: value±error rate. ex) 35±10." | 56 |
| Hydrodynamic size/Surface charge | What is the value of Hydrodynamic size/Surface area of each nanoparticle? Refer to following descriptions. (1,2 same as core size, surface area) 3. If multiple values exist for each material, divide the value using ';', add parentheses after the value, and write the measurement condition in the parentheses. ex) 50 (Solvent: water); 100 (Solvent: medium), 30 (Time: 2 h); 50 (Time: 24 h).  4. If there are both water and culture media conditions, only the culture media condition is selected. However, if there are only conditions taken in water, select conditions taken in water. | -13.1 |
| Measurement method  (e.g., core size) | What measurement method is used for measure core size? Refer to following descriptions. 1. Please use abbreviation form such as TEM, SEM, AFM, XRD, etc. 2. Transmission electron microscopy (TEM) should be selected first, followed by scanning electron microscopy (SEM), atomic force microscopy (AFM), and X-ray diffraction (XRD) in cases where multiple methods are used. | TEM |
| Data source (e.g.,hydrodynamic size) | Is the hydrodynamic size data sourced from the author’s own experiment, manufacturer specifications, reference paper, or is it not provide? Just answer four types: ‘Experiment’, ‘Manufacturer’, ‘Reference’, ‘Not specified’. | Experiment |
| Cell type | Please determine the cell type: whether it is a normal cell or a cancer cell? Refer to following descriptions. 1. Just answer two types: ‘Normal’ or ‘Cancer’.  2. If multiple cell lines were used and their species are different, divide the value using ‘;’ and write cell type followed by its cell line within parentheses. | Cancer (A549); Normal (L929) |
| Cell viability assay | Which cell viability assays were conducted in this paper? Refer to following descriptions. 1. If multiple assays were used, divide the value using ‘;’ 2. Please refer to the following form. ex) CCK-8, MTT, MTS, WST, Alamar blue, CellTiter-Glo, Neural Red, Trypan blue, XTT, Calcein-AM, BrdU, Annexin V PI staining, Hoechst33342, etc. | MTT; MTS; CCK-8 |
| Cell species | What species the cell line originated from? Refer to following description. 1. If multiple cell lines were used and their species are different, divide the value using ‘;’ and write the name of the cell species followed by its cell line within parentheses. 2. Please refer to the following form. ex) Human, Rabbit, Mouse, Pig, etc. | Human (A549); Mouse (L929) |
| Cell organ | What organ the cell line originated from? Refer to following description. 1. If multiple cell lines were used and their organs are different, divide the value using ';' and write the name of the cell species followed by its cell type within parentheses. 2. Please refer to the following form. ex) Lung, Breast, Kidney, Brain, Liver, Bronchial tube, Prostate, Spleen, etc. | Lung (A549); Fibroblast (L929) |

**Table S3.** Data preprocessing methods and algorithms used in different AutoML platforms.

|  | Datasets | Vertex AI | Azure | SageMaker | Dataiku |
| --- | --- | --- | --- | --- | --- |
| Preprocessing | HaHa-Auto | Not visible | StandardScaler; label encoding | Not visible | StandardScaler; Dummy encoding |
|  | HaHa-Manual |  | StandardScaler; label encoding |  | StandardScaler; Dummy encoding |
|  | Ha IIIB |  | StandardScaler; label encoding |  | StandardScaler; Dummy encoding |
|  | HaHa-Auto  (SMOTE) |  | StandardScaler; label encoding |  | StandardScaler; Dummy encoding |
|  | HaHa-Auto  (Filtering) |  | StandardScaler; label encoding |  | StandardScaler; Dummy encoding |
| Algorithm  selection | HaHa-Auto | Neural network | XGBoost | Ensemble^1^ | Gradient Boosted Tress |
|  | HaHa-Manual | Boosted Tree | XGBoost |  | Gradient Boosted Tress |
|  | Ha IIIB | Not applicable | XGBoost |  | Random forest |
|  | HaHa-Auto  (SMOTE) | Neural network | XGBoost |  | Gradient Boosted Tress |
|  | HaHa-Auto  (Filtering) | Neural network | XGBoost |  | Random forest |

^1^SageMaker supports only ensemble models using the following algorithms: XGBoost, LightGBM, Random Forest, Linear models, CatBoost, Extra Trees, Neural Network built using Fast.ai, Neural Network built using PyTorch.

**Table S4.** Comparison of AutoML performance metrics across multiple datasets. Note that the Ha IIIB dataset did not meet the required threshold of 1,000 rows, which is necessary for model training on Vertex AI. Therefore, the metrics for Vertex AI on Ha IIIB are not shown.

|  | Oxide NPs | | | | | | |  |
| --- | --- | --- | --- | --- | --- | --- | --- | --- |
|  |  | HaHa-Auto | HaHa-Manual | Ha IIIB | HaHa-Auto (SMOTE) | HaHa-Auto (Filtering) | Average  ± SD |  |
| Accuracy_N.P._ | Vertex AI | 0.95 | 0.95 | - | 0.89 | 0.96 | 0.94 ± 0.03 |  |
|  | Azure | 0.92 | 0.92 | 0.91 | 0.96 | 0.91 | 0.92 ± 0.02 |  |
|  | SageMaker | 0.94 | 0.93 | 0.92 | 0.97 | 0.94 | 0.94 ± 0.02 |  |
|  | Dataiku | 0.94 | 0.94 | 0.96 | 0.94 | 0.92 | 0.94 ± 0.01 |  |
|  | Average  ± SD | 0.94 ± 0.01 | 0.94 ± 0.01 | 0.93 ± 0.02 | 0.94 ± 0.03 | 0.93 ± 0.02 |  |  |
| F1_N.P._ | Vertex AI | 0.89 | 0.91 | - | 0.89 | 0.89 | 0.90 ± 0.01 |  |
|  | Azure | 0.81 | 0.81 | 0.81 | 0.96 | 0.77 | 0.83 ± 0.07 |  |
|  | SageMaker | 0.87 | 0.84 | 0.85 | 0.97 | 0.85 | 0.88 ± 0.05 |  |
|  | Dataiku | 0.87 | 0.86 | 0.93 | 0.94 | 0.82 | 0.89 ± 0.05 |  |
|  | Average ± SD | 0.86 ± 0.03 | 0.85 ± 0.04 | 0.86 ± 0.05 | 0.94 ± 0.03 | 0.83 ± 0.05 |  |  |
| Precision_N.P._ | Vertex AI | 0.93 | 0.92 | - | 0.92 | 0.89 | 0.92 ± 0.02 |  |
|  | Azure | 0.85 | 0.84 | 0.88 | 0.94 | 0.83 | 0.87 ± 0.04 |  |
|  | SageMaker | 0.90 | 0.87 | 0.87 | 0.96 | 0.88 | 0.90 ± 0.03 |  |
|  | Dataiku | 0.88 | 0.93 | 0.89 | 0.94 | 0.82 | 0.89 ± 0.05 |  |
|  | Average ± SD | 0.89 ± 0.03 | 0.89 ± 0.04 | 0.88 ± 0.01 | 0.94 ± 0.01 | 0.86 ± 0.03 |  |  |
| Recall_N.P._ | Vertex AI | 0.85 | 0.89 | - | 0.86 | 0.89 | 0.87 ± 0.02 |  |
|  | Azure | 0.78 | 0.78 | 0.76 | 0.97 | 0.71 | 0.80 ± 0.10 |  |
|  | SageMaker | 0.85 | 0.82 | 0.83 | 0.98 | 0.85 | 0.87 ± 0.06 |  |
|  | Dataiku | 0.86 | 0.80 | 0.97 | 0.94 | 0.82 | 0.88 ± 0.07 |  |
|  | Average ± SD | 0.84 ± 0.03 | 0.83 ± 0.08 | 0.83 ± 0.05 | 0.93 ± 0.05 | 0.82 ± 0.08 |  | |

**Table S5.** Comparison of the applicability domain (AD) of models trained on HaHa-Auto and Ha IIIB datasets for categorical attributes. The number in parentheses represents the number of components included in each dataset.

| Attributes | Datasets | Components |
| --- | --- | --- |
| Material type | HaHa-Auto (23) | Ag_2_O, Al_2_O_3_, AlOOH, CeO_2_, CO_3_O_4_, CoO, CuO, Fe_2_O_3_, Fe_3_O_4_, GO, HfO_2_, Mn_2_O_3_, Mn_3_O_4_, MgO, NiO, SiO_2_, SnO_2_, TiO_2_, V_2_O_5_, WO_3_, Y_2_O_3_, ZnO, ZrO_2_ |
|  | Ha IIIB (12) | Al_2_O_3_, CO_3_O_4_, CoO, Cr_2_O_3_, CuO, Fe_2_O_3_, Fe_3_O_4_, In_2_O_3_, Mn_2_O_3_, SiO_2_, TiO_2_, ZnO |
| Cell viability assay | HaHa-Auto (21) | Alamar blue, ATP, Annexin V/PI staining, C11-BODIPY, CCK-8, CellTiter-Glo, CFA, Coomassie Blue, FDA uptake, LDH, Live/Dead, MTS, MTT, Neutral red, Presto Blue, Resazurin, SRB, Trypan blue, TTC, WST, XTT |
|  | Ha IIIB (10) | ATP, Annexin V/PI staining, CCK-8, CellTiter-Glo, CytoTox-Glo, LDH, MTS, MTT, RTCA, WST |
| Cell organ | HaHa-Auto (24) | Adrenal gland, Aorta, Blood, Bone, Brain, Breast, Cervix, Colon, Embryo, Fibroblast, Heart, Kidney, Liver, Lung, Muscle, Nasal septum, Ovary, Pancreas, Prostate, Skin, Testis, Tongue, Umbilical artery, Umbilical vein |
|  | Ha IIIB (5) | Blood, Liver, Lung, Nose, Skin |
| Cell species | HaHa-Auto (5) | Chicken, Hamster, Human, Monkey, Mouse |
|  | Ha IIIB (3) | Human, Hamster, Mouse |
